# Impaired exercise outcomes with significant bronchodilator responsiveness in children with prematurity‐associated obstructive lung disease

**DOI:** 10.1002/ppul.26019

**Published:** 2022-06-14

**Authors:** Michael Cousins, Kylie Hart, E. Mark Williams, Sailesh Kotecha

**Affiliations:** ^1^ Department of Child Health Cardiff University School of Medicine Cardiff UK; ^2^ Faculty of Life Sciences and Education University of South Wales Pontypridd UK

**Keywords:** albuterol, bronchopulmonary dysplasia, exercise test, premature birth, respiratory function tests

## Abstract

**Introduction:**

Preterm‐born children have their normal in‐utero lung development interrupted, thus are at risk of short‐ and long‐term lung disease. Spirometry and exercise capacity impairments have been regularly reported in preterm‐born children especially those who developed chronic lung disease of prematurity (CLD) in infancy. However, specific phenotypes may be differentially associated with exercise capacity. We investigated exercise capacity associated with prematurity‐associated obstructive (POLD) or prematurity‐associated preserved ratio of impaired spirometry (pPRISm) when compared to preterm‐ and term‐controls with normal lung function.

**Materials and Methods:**

Preterm‐ and term‐born children identified through home screening underwent in‐depth lung function and cardiorespiratory exercise testing, including administration of postexercise bronchodilator, as part of the Respiratory Health Outcomes in Neonates (RHiNO) study.

**Results:**

From 241 invited children, aged 7–12 years, 202 underwent exercise testing including 18 children with POLD (percent predicted (%)FEV_1_ and FEV_1_/FVC < LLN); 12 pPRISm (%FEV_1_ < LLN and FEV_1_/FVC ≥ LLN), 106 preterm‐controls (PT_c_, %FEV_1_ ≥ LLN) and 66 term‐controls (T_c_, %FEV_1_ > 90%). POLD children had reduced relative workload, peak O_2_ uptake, CO_2_ production, and minute ventilation compared to T_c_, and used a greater proportion of their breathing reserve compared to both control groups. pPRISm and PT_c_ children also had lower O_2_ uptake compared to T_c_. POLD children had the greatest response to postexercise bronchodilator, improving their %FEV_1_ by 19.4% (vs 6.3%, 6% 6.3% in pPRISm PT_c,_T_c_, respectively; *p* < .001).

**Conclusion:**

Preterm‐born children with obstructive airway disease had the greatest impairment in exercise capacity, and significantly greater response to postexercise bronchodilators. These classifications can be used to guide treatment in children with POLD.

## 1 INTRODUCTION

It is now recognized that preterm‐born children are at greater risk of future respiratory health sequelae, both in the short‐^1^ and long‐term,^2^ when compared to term‐born infants. Since the etiology is multifactorial, including a combination of immature lungs exposed to ante‐ and postnatal inflammatory processes due to injurious exposures,^1^ it is very likely that different phenotypes may result from these early life exposures.^3^ Those most at risk are those who develop chronic lung disease of prematurity (CLD, also known as bronchopulmonary dysplasia or BPD) in infancy. However, it is increasingly recognized that even those who are born late preterm are also at risk of future lung disease.^4^

Although it is anticipated that low function may lead to reduced exercise capacity, the results are often variable,^5^ as shown by our systematic review of publications reporting minimal differences in exercise capacity in preterm‐born children with and without CLD compared to their term‐born counterparts^5^. When preterm‐born subjects without CLD and those with CLD were compared to term groups, ~5% and ~10% differences for peak oxygen uptake were noted. Furthermore, although we recently showed improvement in percent predicted forced expiratory volume in 1 s (%FEV_1_) after combined inhaled corticosteroids and long‐acting β_2_‐agonists treatment, exercise capacity did not improve after 12‐weeks of treatment.^6^ It is unclear which specific groups of preterm‐born children with decreased lung function will respond to such therapy.

Additionally, reporting of exercise‐induced bronchoconstriction for preterm‐born children is variable with not all studies showing bronchoconstriction after exercise. Furthermore, the response of exercise‐induced bronchoconstriction to bronchodilator is also variable. Joshi et al. showed that exercise resulted in an 11.1% decrease in %FEV_1_ after exercise in preterm‐born children with CLD, which responded to bronchodilator with the %FEV_1_ increasing by 16% absolute value. In contrast, the study by Nixon et al resulted in only a 2% decrease in %FEV_1_ after exercise in two groups of preterm children born with very low birth weight.^7^

Taken together, these variable observations may be due to different populations, early life exposures and interventions, different assessment methods used or, indeed, preterm‐born children may have adequate exercise capacity. An additional plausible explanation is that different phenotypes of lung disease may be responsible for these variable results. Thus, it is important to identify pragmatic methods that can be used in the outpatient clinic, that will direct therapy for these children who are now considered at risk of premature development of chronic obstructive pulmonary disease (COPD).^8^

While obstructive airway disease is well‐defined, seen in multiple disease states including asthma, cystic fibrosis, and COPD,^9^ harder to categorize are those with impaired lung function but normal FEV_1_/forced vital capacity (FVC) ratio (FEV_1_/FVC). While these may include restrictive lung disease, this requires total lung capacity to diagnose,^10^ which is not practical in the majority of clinical settings. The concept of preserved ratio of impaired spirometry (PRISm) has been explored recently in adult populations, associating decreased FEV_1_ but normal/increased FEV_1_/FVC with COPD, cardiovascular disease, and all‐cause mortality.^11^, ^12^ However, this has been poorly studied in pediatric populations, including preterm‐born children.

We, therefore, identified preterm‐born children with prematurity‐associated obstructive lung disease (POLD, %FEV_1_ < lower limit of normal (LLN) and FEV_1_/FVC < LLN) and prematurity‐associated PRISm (pPRISm, FEV_1_ < LLN and FEV_1_/FVC ≥ LLN) from our Respiratory Health Outcomes in Neonates (RHiNO) cohort and compared their results with preterm‐ (preterm controls—PT_c_, FEV_1_ ≥ LLN) and term‐born recruits (term controls—T_c_) with normal lung function. Our aim was to assess their baseline exercise capacity, postexercise bronchoconstriction, and postexercise bronchodilator responses, along with their static lung volumes.

## 2 MATERIALS AND METHODS

### 2.1 Study population and testing

Children born preterm between 2005 and 2011, along with term controls, were prospectively recruited as part of the RHiNO study (EudraCT: 2015‐003712‐20).^6^, ^13^ These children were identified during a previous questionnaire study,^14^, ^15^ and were recruited to participate in the current study, which ran between January 2017 and August 2019. Inclusion criteria were gestational age at birth ≤34 weeks′ gestation for preterm‐born children and at ≥37 weeks′ gestation for term‐born children, age 7–12 years, and geographically accessible. Children with significant congenital, cardiac or neurodevelopmental abnormalities were excluded. Recruitment was postponed in children with a recent (within the past 3 weeks) respiratory tract infection.

After the invitation, and after obtaining informed written consent/assent from the parent(s)/child, a screening visit at home which included spirometry (MicroLoop; Vyaire) was completed. Children with %FEV_1_ ≤ 85% were subsequently invited to attend the Children and Young Adults Research Unit at Cardiff, United Kingdom, for further in‐depth lung function testing with the specific aim of recruitment into our double‐blinded, randomized, placebo‐controlled trial of inhaled corticosteroids alone or combined with long‐acting beta‐2 agonists.^6^ If they were noted to have %FEV_1_ of >85% at the children′s hospital visit they were not entered into the randomized control trial but were included as preterm controls who had %FEV_1_ > 85% if they had previously been identified as potential preterm controls (see Supporting Information for further details). Preterm‐born children with %FEV_1_ > 85% and term children with %FEV_1_ > 90% were studied in parallel as control groups. After obtaining further written informed consent/assent from the parent(s)/child, the children underwent detailed spirometry, assessment of their exercise capacity including responses to administration of postexercise bronchodilator, measurement of fractional exhaled nitric oxide (FE_NO_) and skin prick testing, as described in detail in the Supporting Information. Briefly, spirometry was performed using the MasterScreen Body and PFT systems with SentrySuite measurement software version 2.17 (Vyaire Medical) according to ERS/ATS guidelines^16^ and normalized against the Global Lung Initiative reference values.^17^ A minimum of three tests were performed, aiming for the intratest criteria recommended by Miller et al.^16^ Spirometry was stopped once satisfactory testing was obtained, or if the child was unable to or did not wish to continue. Blinded QC was performed on all measurements. Spirometry was conducted at baseline, at 5–10, 15–20, 25–30, and 40–45 min after exercise, and 15 min after administration of postexercise bronchodilator (400 μg of albuterol via a spacer device). Children were excluded if they were unable to perform adequate spirometry or exercise. Any respiratory medicines including inhalers were withheld as described in the Supporting Information.

Cardiopulmonary exercise testing using a cycle ergometer started with a rest phase to obtain baseline readings, followed by 3 min of minimally loaded cycling, followed by a ramp increasing 1 Watt every 6 s until cadence could no longer be consistently maintained greater than 60 rpm, completing with 2 min of minimally loaded pedaling at the end of the test. A maximal test was considered if two of the following four criteria were achieved: Respiratory Exchange Ratio (RER) > 1.00; heart rate ≥ 80% predicted (220 bpm—age); ≥9/10 on OMNI scale (pictorial scale for rating of perceived exertion)^18^; peak oxygen uptake (V̇O_2_) plateau based on visual analysis.

Perinatal demographics and diagnoses of diseases during the neonatal admission and beyond were obtained from NHS Wales Informatics Service records and from case‐note review of neonatal medical records, including CLD defined as a combination of 28 days of age and/or 36 weeks′ postmenstrual age in those born ≤32 weeks′ gestation or at ≥56 days of age in those born at >32 weeks. Data on respiratory history was parent‐reported during history‐taking and with a validated respiratory questionnaire^19^ at the home visit.

Spirometry results were used to formally classify the children into the four groups, namely POLD (FEV_1_ < LLN, FEV_1_/FVC < LLN), pPRISm (FEV_1_ < LLN, FEV_1_/FVC ≥ LLN), PT_c_ (FEV_1_ ≥ LLN), and T_c_ (%FEV_1_ > 90%) groups. Postexercise bronchodilator reversibility was defined as >10% absolute change in %FEV_1_.

### 2.2 Ethical approval

Ethics approval was granted by the South West Central Bristol Ethics Committee (Ref 15/SW/0289). Parents and children (where possible) provided written informed consent/assent for the home and hospital visit separately.

### 2.3 Statistical analysis

One‐way analysis of variance (ANOVA) with Bonferroni correction was used for multiple groups comparisons at baseline. Categorical data were assessed using Pearson′s *χ* ^2^ tests. Within‐group (baseline, postexercise and postexercise bronchodilator) and between‐group (preterm and term groups) comparisons across timepoints were compared using two‐way repeat measures ANOVA. Change scores between groups were compared using one‐way ANOVA. All group comparisons were corrected for multiplicity using Bonferroni correction. Statistical analysis was performed using SPSS version 26 (IBM). A *p* value of <0.05 was considered significant.

## 3 RESULTS

### 3.1 Participants

**Figure 1:**
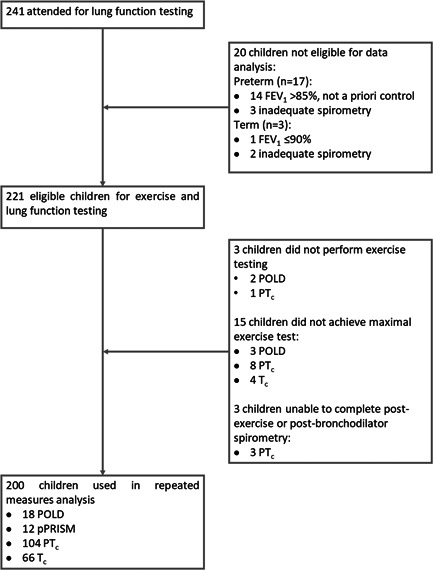
Flow diagram outlining recruitment numbers for lung function testing and numbers for those included in final analysis.

As shown in Figure 1, from 241 children who attended for testing, 20 of these children were excluded: 5 children were unable to perform adequate spirometry on the day (3 preterm, 2 term); 14 children who had %FEV_1_ ≤ 85% at screening achieved %FEV_1_ of >85% at the children′s hospital visit, and 1 term child was excluded due to FEV_1_ ≤ 90%. Twenty‐one POLD, 12 pPRISm, 115 PT_c_, and 70 T_c_ children performed exercise testing. Fifteen children did reach maximal exercise (3 POLD, 8 PT_c_, 4 T_c_), and a further 3 PT_c_ children could not complete satisfactory spirometry at all three timepoints and thus were excluded from the repeated measures ANOVA analysis. Eighteen POLD, 12 pPRISm, 104 PT_c_, and 66 T_c_ were included in the repeated measures analysis.

Participant demographics (Table 1) showed POLD children were born earlier and were smaller than PT_c_ children (29.4 vs. 30.9 weeks′ gestation and 1320 vs. 1690 g). There were no differences between other perinatal details between preterm groups. POLD children had higher rates of asthma (35% vs. 7%), wheeze ever (91% vs. 27%), recent wheeze (44% vs. 13%), and albuterol use (35% vs. 6%) compared to term controls respectively, with 35% of POLD children demonstrating atopy on skin prick testing compared to 11% of T_c_ children. There were no differences between the preterm groups within these characteristics. FE_NO_ was 50% greater in the POLD group (33.1 vs. 21.8 vs. 21.6 vs. 19.9 ppb for pPRISm, PT_c_, T_c_, respectively), but this was not statistically significant.

**Table 1:** Baseline characteristics of participants including anthropometric, perinatal, respiratory/atopy details, and fractional exhaled nitric oxide (FE_NO_) results for preterm obstructive lung disease (POLD), preterm preserved ratio of impaired spirometry (pPRISm), preterm (PT_c_) and term (*T*_c_) controls.

	POLD (*n* = 23)	pPRISm (*n* = 12)	PT_c_ (*n* = 116)	T_c_ (*n* = 70)
*Current demographics*				
Age, years	10.7 (10.1–11.2)	11.2 (10.3– 12.0)	**11.1 (10.8**–**11.3)** ₴₴	10.5 (10.2–10.7)
Male, *n* (%)	11 (48%)	3 (25%)	59 (51%)	37 (53%)
Height, cm	141.7 (136.8–146.5)	144.1 (135.1–153.1)	146.5 (144.7–148.2)	143.9 (141.6–146.1)
Height, *Z*‐score	−0.06 (−0.55 to 0.43)	−0.15 (−1.16 to 0.85)	0.34 (0.17–0.50)	0.48 (0.25–0.72)
Weight, kg	36.9 (32.0–41.8)	38.0 (28.2–47.9)	39.8 (38–41.6)	37.9 (35.4–40.4)
Weight, *Z*‐score	0.08 (−0.51 to 0.66)	−0.37 (−1.61 to 0.88)	0.38 (0.20–0.57)	0.46 (0.22–0.71)
BMI, kg/m^2^	18.0 (16.5–19.5)	17.6 (14.8–20.3)	18.4 (17.7–19.0)	18.0 (17.3–18.8)
BMI, *Z*‐score	0.13 (−0.47 to 0.73)	−0.40 (−1.53 to 0.73)	0.23 (0.00–0.46)	0.30 (0.05–0.56)
*Perinatal demographics*				
Gestation, decimal weeks	**29.4 (28.1**–**30.6)** †‡‡‡	**29.8 (28.0**–**31.7)** ¶¶¶	**30.9 (30.4**–**31.4)** ₴₴₴	40.0 (39.7–40.3)
Birth weight, g	**1320 (1083**–**1557)** †‡‡‡	**1405 (993**–**1817)** ¶¶¶	**1690 (1585**–**1795)** ₴₴₴	3528 (3404–3651)
Birth weight, *Z*‐score	−0.27 (−0.86 to 0.33)	−0.26 (−1.19 to 0.67)	0.21 (−0.04 to 0.46)	0.08 (−0.15 to 0.31)
IUGR, *n* (%)	5 (22%)	3 (25%)	19 (16%)	4 (6%)
Antenatal maternal corticosteroids, *n* (%)	**20 (87%)** ‡‡‡	**11 (92%)** ¶¶¶	**97 (84%)** ₴₴₴	0 (0%)
Invasive ventilation, *n* (%)	**14 (61%)** ‡‡‡	**4 (33%)** ¶¶¶	**47 (41%)** ₴₴₴	0 (0%)
CLD, *n* (%)	**10 (44%)** ‡‡‡	**3 (25%)** ¶¶¶	**25 (22%)** ₴₴₴	0 (0%)
*Respiratory history*				
Doctor‐diagnosed asthma, *n* (%)	**8 (35%)** ‡‡	3 (25%)	23 (20%)	5 (7%)
Wheeze ever, *n* (%)	**21 (91%)** †††	7 (58%)	**55 (47%)** ₴	19 (27%)
Recent wheeze, *n* (%)	**10 (44%)** ‡	2 (17%)	24 (21%)	9 (13%)
Current salbutamol use, *n* (%)	**8 (35%)** ‡‡	1 (8%)	20 (17%)	4 (6%)
Current maternal smoking, *n* (%)	**4 (17%)** ‡‡	**2 (17%)** ¶¶	9 (8%)	0 (0%)
*Skin prick testing*				
≥1 positive test(s), *n* (%)	**8 (35%)** ‡	3 (25%)	30 (26%)	8 (11%)

FE_NO_	**(*n*** = **23)**	**(*n*** = **12)**	**(*n*** = **113)**	**(*n*** = **68)**
FE_NO_ > 35ppb, *n* (%)	9/22 (41%)	2/12 (17%)	20/113 (18%)	8/68 (12%)
Highest FE_NO_, ppb	33.1 (20.7–45.6)	21.8 (3.70–40.0)	21.6 (17.8–25.4)	19.9 (15.3–24.4)

*Note*: Results expressed as mean and 95% confidence intervals for continuous data (one‐way ANOVA with Bonferroni correction) or number and % proportion (Pearson′s *χ* ^2^ test) unless otherwise specified.

Significance symbols: *POLD versus pPRISm, ^†^POLD versus PT_c_, ^‡^POLD versus T_c_, ^¥^pPRISm versus PT_c_, ^¶^PRISm versus T_c_, ^₴^PTc versus T_c_. (Single symbol denotes significance level <0.05, double symbol <0.01, triple symbol <0.001).

Abbreviations: ANOVA, analysis of variance; BMI, body mass index; CLD, chronic lung disease of prematurity; FE_NO_, fractional exhaled nitric oxide; IUGR, intrauterine growth restriction.

### 3.2 Baseline lung function and exercise testing

All four groups achieved comparable peak heart rate suggesting a similar degree of exertion was performed (Table 2). When compared to the T_c_ group, POLD children had deficits in relative workload (2.16 vs. 2.72 W/kg), peak O_2_ uptake (31.8 vs. 38.1 ml/kg/min), CO_2_ production (35.3 vs. 43.7 ml/kg/min), and minute ventilation V̇E, both as a whole (48 vs. 59 L/min) and relative to birth weight (1.26 vs. 1.61 L/kg/min). V̇E relative to height was lower for POLD compared to both preterm and term controls (0.33 vs. 0.39 vs. 0.42 L/m/min, respectively). Additionally, POLD children used up a greater proportion of their respiratory reserve than both controls (6.5 vs. 24.8 vs, 24.8% compared to PT_c_ and T_c_). pPRISm and PT_c_ groups also demonstrated lower peak O_2_ uptake compared to the T_c_ group. pPRISm had a trend towards lower values, not dissimilar to POLD, but did not reach statistical significance possibly due to small numbers available for study.

**Table 2:** Cardiopulmonary exercise testing and body plethysmography results for preterm obstructive lung disease (POLD), preterm preserved ratio of impaired spirometry (pPRISm), preterm (PT_c_), and term (T_c_) controls.

**Exercise**	**POLD (*n*** = **18)**	**pPRISm (*n*** = **12)**	**PT**_**c **_ **(*n*** = **107)**	**T**_**c **_ **(*n*** = **66)**
Peak heart rate, bpm	186.8 (181.2–192.3)	190.8 (182.9–198.6)	189.7 (187.5–191.9)	189.7 (187.2–192.3)
Peak respiratory rate, brpm	63.3 (58.2–68.4)	63.6 (57.4–69.8)	62.2 (60.1–64.4)	66.3 (64.0–68.6)
Workload, W/kg	**2.16 (1.90**–**2.42)** ‡‡	2.30 (1.93–2.67)	**2.44 (2.33**–**2.56)** ₴	2.72 (2.58–2.86)
Peak O_2_ uptake, ml/kg/min	**31.8 (28.6**–**35.0)** ‡	**30.3 (26.6**–**34.0)** ¶	**33.7 (32.3**–**35.2)** ₴₴	38.1 (36.1–40.1)
Peak CO_2_ production, ml/kg/min	**35.3 (31.2**–**39.3)** ‡‡	36.9 (32.3–41.4)	40.3 (38.5–42.0)	43.7 (41.6–45.8)
Peak V̇E, L/min	**48.0 (41.8**–**54.2)** ‡	50.4 (39.2–61.5)	58.1 (55.1–61.1)	59.0 (55.1–62.8)
Relative peak V̇E, L/kg/min	**1.26 (1.12**–**1.39)** ‡‡	1.32 (1.14–1.51)	1.48 (1.41–1.56)	1.61 (1.53–1.70)
Peak V̇E vs height, L/m/min	**0.33 (0.30**–**0.36)** †‡‡	0.34 (0.28–0.40)	0.39 (0.38–0.41)	0.41 (0.39–0.43)
Highest RER	**1.14 (1.10**–**1.19)** **†††	1.27 (1.21–1.32)	**1.24 (1.22**–**1.25)** ₴	1.20 (1.18–1.22)
Breathing reserve max, %	**6.5 (−1.8 to 14.8)** †††‡‡‡	19.4 (11.3–27.5)	24.8 (21.6–28.0)	24.8 (21.8–27.8)

**Static lung volumes (plethysmography)**	**(*n*** = **20)**	**(*n*** = **11)**	**(*n*** = **110)**	**(*n*** = **68)**
FRC, *Z*‐score	**0.66 (0.20**–**1.12)** ***†††‡‡‡	**−1.18 (−1.71 to −0.66)** ¥¥¶¶	−0.30 (−0.44 to −0.16)	−0.17 (−0.36 to 0.02)
%predicted FRC	**115.6 (105**–**126.3)** ***†††‡‡‡	**78.4 (69.1**–**87.7)** ¥¥¶¶	94.8 (92.0–97.5)	97.5 (93.6–101.4)
TLC, *Z*‐score	−0.04 (−0.43 to 0.35)	**−1.64 (−2.01 to −1.27)** ***¥¥¥¶¶¶	**−0.29 (−0.43 to −0.15)** ₴₴	0.11 (−0.05 to 0.27)
%predicted TLC	99.7 (94.4–105.0)	**79.1 (74.8**–**83.4)** ***¥¥¥¶¶¶	**96.3 (94.4**–**98.1)** ₴₴	101.6 (99.4–103.8)
RV, *Z*‐score	**0.49 (0.17**–**0.80)** ***†††‡‡‡	−0.48 (−0.73 to −0.24)	−0.25 (−0.35 to −0.15)	−0.27 (−0.41 to −0.14)
%predicted RV	**127.3 (109.6**–**145.0)** ***†††‡‡‡	79.1 (68.9–89.3)	90.3 (85.7–94.9)	89.1 (83.1–95.2)
RV/TLC	**0.30 (0.27**–**0.33)** **†††‡‡‡	0.23 (0.21–0.25)	0.22 (0.21–0.23)	0.21 (0.19–0.22)
RV/TLC, *Z*‐score	**0.94 (0.65**–**1.24)** **†††‡‡‡	0.18 (−0.07 to 0.44)	0.04 (−0.07 to 0.16)	−0.10 (−0.24 to 0.03)
Proportion RV/TLC > 0.30, *n* (%)	**8/20 (40%)** †††‡‡‡	0/11 (0%)	9/110 (8%)	4/68 (6%)
Proportion %TLC < LLN, *n* (%)	0/20 (0%)	**5/11 (46%)** ***¥¥¥¶¶¶	5/110 (5%)	0/68 (0%)

*Note*: Results expressed as mean and 95% confidence intervals for continuous data (one‐way ANOVA with Bonferroni correction) or number and % proportion (Pearson′s *χ* ^2^ test) unless otherwise specified.

Significance symbols: *POLD versus pPRISm, ^**†**^POLD versusPT_c_, ^**‡**^POLD versus T_c_, ^**¥**^pPRISm versus PT_c_, ^**¶**^PRISm versus T_c_, ^**₴**^PT_c_ versus T_c_. (Single symbol denotes significance level <0.05, double symbol <0.01, triple symbol p<0.001).

Abbreviations: ANOVA, analysis of variance; bpm, beats per minute; brpm, breaths per minute; FRC, functional residual capacity; LLN, lower limit of normal; RER, respiratory exchange ratio; TLC, total lung capacity; RV, residual volume; V̇E, minute ventilation.

### 3.3 Lung volumes

**Figure 2:**
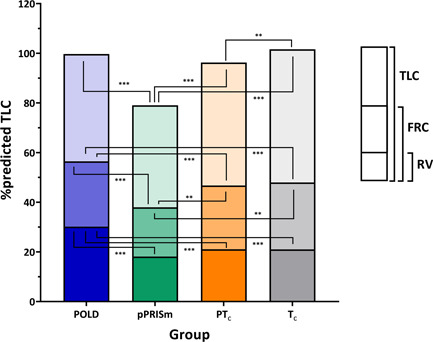
Graph representing static lung volumes from body plethysmography, with percent predicted total lung capacity (%predicted TLC) as the overall size of the bar, and functional residual capacity (FRC) and residual volume (RV) as proportions of the TLC, for preterm obstructive lung disease (POLD), preterm preserved ratio of impaired spirometry (pPRISm), preterm (PT_c_) and term (T_c_) controls. Color figure can be viewed at wileyonlinelibrary.com

Static lung volume testing (Table 2 and Figure 2) demonstrated different patterns for the POLD and pPRISm groups. POLD children had increased FRC (116% predicted), normal TLC (100% predicted), and high RV (127% predicted), with an average RV/TLC ratio of 0.3; whereas pPRISm group had low FRC (78% predicted) and TLC (79% predicted). Forty‐six percent of the pPRISm group had TLC < LLN, consistent with true restrictive lung disease, significantly higher than all the other groups.

### 3.4 Spirometry

**Figure 3:**
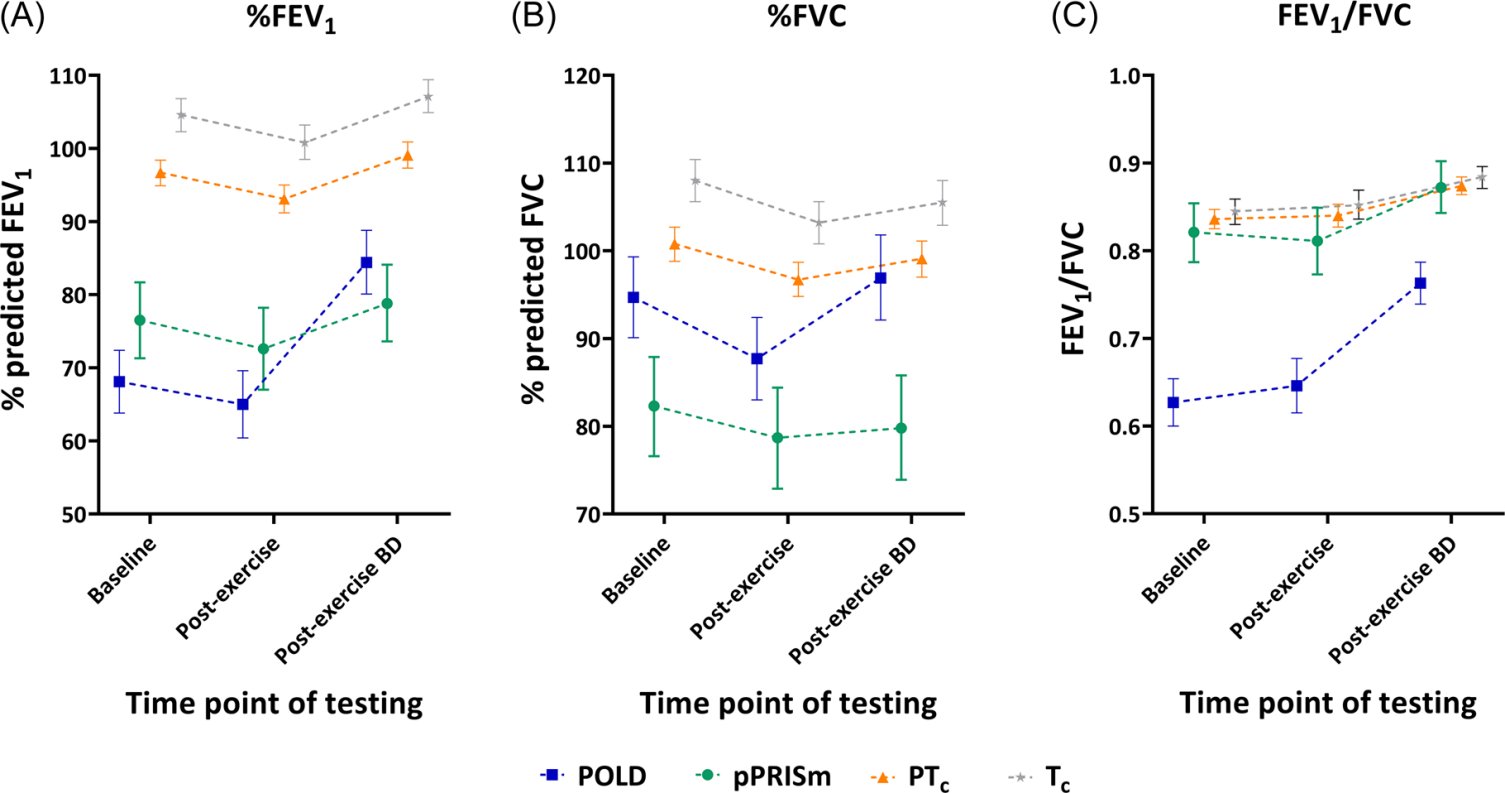
Graph representing percent predicted forced expiratory volume in 1 s (%FEV_1_), percent predicted forced vital capacity (%FVC) and FEV_1_/FVC ratio at baseline, postexercise and postexercise bronchodilator, for preterm obstructive lung disease (POLD), preterm preserved ratio *of* impaired spirometry (pPRISm), preterm (PT_c_) and term (T_c_) controls. [Color figure can be viewed at wileyonlinelibrary.com]

As the groups were classified according to their spirometry, as expected %FEV_1_ was lowest in the POLD and pPRISm groups (68% vs. 77% vs. 97% vs. 105% predicted for POLD, pPRISm, PT_c,_ and T_c_), as per Table 3 and Figure 3. FVC was also lower in POLD (95%) but especially pPRISm (82%) compared to both controls (101% and 108% for PT_c_ and T_c_). Again, as per how groups were divided, FEV_1_/FVC ratio was lowest in POLD children.

**Table 3:** Spirometry parameters at baseline and response to exercise and Postexercise bronchodilator therapy for preterm obstructive lung disease (POLD), preterm preserved ratio of impaired spirometry (pPRISm), preterm (PT_c_), and term (T_c_) controls.

	**POLD (*n*** = **18)**	**pPRISm (*n*** = **12)**	**PT**_**c **_ **(*n*** = **104)**	**T**_**c **_ **(*n*** = **66)**
*FEV*_1_				
Baseline, % predicted	**68.1 (63.8–72.4)** †††‡‡‡	**76.5 (71.3–81.7)** ¥¥¥¶¶¶	**96.7 (94.9–98.4)** ₴₴₴	104.6 (102.3**–**106.8)
Postexercise, % predicted	^**∂**^ **65.0 (60.4–69.6)** †††‡‡‡	^**∂∂**^ **72.6 (67.0–78.2)** ¥¥¥ ¶¶¶	^**∂∂∂**^ **93.1 (91.2–95.0)** ₴₴₴	^∂∂∂^100.8 (98.5**–**103.2)
Postexercise bronchodilator, % predicted	^**₸₸₸**^ **84.4 (80.1–88.8)** †††‡‡‡	^**₸₸₸**^ **78.8 (73.6–84.1)** ¥¥¥¶¶¶	^**₸₸₸**^ **99.1 (97.3–100.9)** ₴₴₴	^₸₸₸^107.1 (104.9**–**109.4)
Baseline to Postexercise change, % predicted	−3.1 (−6.0 to −0.2)	−3.9 (−6.6 to −1.2)	−3.5 (−4.3 to −2.8)	−3.7 (−5.0 to −2.5)
Postexercise to postexercise bronchodilator change, % predicted	**19.4 (15.0–23.9)** ***†††‡‡‡	6.3 (2.7**–**9.8)	6.0 (5.1**–**6.9)	6.3 (5.0**–**7.6)
Bronchodilator response >10% FEV_1_, *n* (%)	**17/18 (94%)** ***†††‡‡‡	4/12 (33%)	21/104 (20%)	11/66 (17%)
*FVC*				
Baseline, % predicted	**94.7 (90.1–99.3)** **‡‡‡	**82.3 (76.6–87.9)** ¥¥¥¶¶¶	**100.8 (98.8–102.7)** ₴₴₴	108.0 (105.6**–**110.4)
Postexercise, % predicted	^∂∂∂^ **87.7 (83.0–92.4)** ††‡‡‡	^∂^ **78.7 (72.9–84.4)** ¥¥¥¶¶¶	^∂∂∂^ **96.7 (94.8–98.7)** ₴₴₴	^∂∂∂^103.2 (100.8**–**105.6)
Postexercise bronchodilator, % predicted	^₸₸₸^ **96.9 (92.1–101.8)** ***‡	**79.8 (73.9–85.8)** ***¥¥¥¶¶¶	^₸₸₸^ **99.1 (97.0–101.1)** ₴₴	^₸₸₸^105.5 (102.9**–**108.0)
Baseline to postexercise change, % predicted	**−7.0 (−10.1 to −3.9)** †	−3.6 (−5.7 to −1.5)	−4.0 (−4.9 to −3.2)	−4.8 (−5.7 to −3.8)
Postexercise to postexercise bronchodilator change, % predicted	**9.2 (5.2 to 13.3)** ***†††‡‡‡	1.2 (−0.3 to 2.6)	2.3 (1.4**–**3.2)	2.3 (1.4**–**3.2)
*FEV*_1_ */FVC*				
Baseline, ratio	**0.627 (0.600–0.654)** ***†††‡‡‡	0.821 (0.787**–**0.854)	0.836 (0.825**–**0.847)	0.845 (0.830**–**0.859)
Postexercise, ratio	**0.646 (0.615–0.677)** ***††† ‡‡‡	0.811 (0.773**–**0.849)	0.840 (0.827**–**0.853)	0.852 (0.836**–**0.869)
Postexercise bronchodilator, ratio	^₸₸₸^ **0.763 (0.739–0.787)** ***†††‡‡‡	^₸₸₸^0.872 (0.843**–**0.902)	^₸₸₸^0.874 (0.864**–**0.884)	^₸₸₸^0.884 (0.871**–**0.896)
Baseline to postexercise change	0.019 (−0.003 to 0.042)	−0.010 (−0.033 to 0.013)	0.004 (−0.002 to 0.011)	0.008 (−0.001 to 0.017)
Postexercise to postexercise bronchodilator change	**0.116 (0.086–0.147)** **†††‡‡‡	0.062 (0.032**–**0.091)	0.033 (0.026**–**0.041)	0.031 (0.023**–**0.040)

*Note*: Results expressed as mean and 95% confidence intervals for continuous data (two‐way ANOVA with Bonferroni correction).

Significance symbols: *POLD versus pPRISm, ^†^POLD versus PT_c_, ^‡^POLD versus T_c_, ^¥^pPRISm versus PT_c_, ^¶^PRISm versus T_c_, ^₴^PTc versus T_c_; ^∂^Baseline versus postexercise,^₸^Postexercise versus postbronchodilator. (Single symbol denotes significance level <0.05, double symbol <0.01, triple symbol <0.001).

Abbreviations: ANOVA, analysis of variance; FEV1, forced expiratory volume in 1  s; FVC, forced vital capacity.

After exercise, there was a similar drop of 3%–4%FEV_1_ in all groups; however, POLD children had a significantly greater drop in %FVC of 7% compared to 4% in the PT_c_ children.

Following postexercise bronchodilator administration, mean %FEV_1_ increased by 19% in the POLD children, with 94% of these children demonstrating reversibility. There was a mean increase of ~6% for %FEV_1_ for all the other groups following postexercise bronchodilator administration. There was also a significantly greater increase in %FVC and FEV_1_/FVC in the POLD group when compared to all the other groups.

## 4 DISCUSSION

It is increasingly recognized that CLD diagnosed in infancy is not a good predictor of future lung function deficits^13^ thus we need to identify preterm‐born children who have significant lung function decrements to establish why they continue to have respiratory deficits in childhood and beyond, and importantly to identify optimal treatments. Therefore, we identified preterm‐born children with low lung function defined as %FEV_1_ of less than the LLN, and further classified them into those with low (obstructive phenotype) or normal/increased (PRISm) FEV_1_/FVC ratios to assess their exercise capacity. We noted that the POLD group had lower exercise capacity when compared to their term‐born counterparts who had normal lung function. Additionally, the POLD group had significant reversible postexercise bronchodilator responses with 94% having a > 10% increase in %FEV whereas only 6% of the pPRISM and both control groups did. Interestingly, the POLD group also had a trend toward higher levels of FE_NO_ when compared to the pPRISm and both control groups.

The POLD children had greater rates of respiratory symptoms with over 90% having experienced wheeze, but only a third had been diagnosed with asthma or were currently using a bronchodilator, suggesting that many preterm‐born children with lung function abnormalities are not being identified and are not adequately treated. The rate of positive skin prick test was also approximately a third, and FE_NO_ of >35 ppb (the suggested cut off for association with eosinophilic asthma)^20^ was noted in approximately 40% of the POLD group. Preterm‐born children are often (mis)classified as having asthma with higher rates of diagnosis when compared to term‐born peers.^21^ Atopy has not been shown to be associated with wheezing in preterm‐born children,^15^, ^22^ including those specifically with BPD,^23^ but while some children will have classical asthma (possibly those with the positive SPT and raised FE_NO_), the majority are likely to have a different underlying disease process. Pulmonary inflammation in this group is more likely than in the non‐obstructive pPRISm group as FE_NO_, which has been shown not to be increased in previous studies,^24^ was trending toward higher levels in the obstructive group, but was similar in the pPRISm and preterm and term control groups. It is feasible that early life factors such as chorioamnionitis, gestational age, and intrauterine growth restriction are associated with subsequent lung disease in preterm‐born infants.^13^, ^25^ However, our data suggest that different phenotypes of lung disease may result from these different early life exposures.

On maximal exercise, the POLD and pPRISm groups had 6.3 and 7.8 ml/kg/min lower peak V̇O_2_ than the *T*_c_ group, which is much greater than the 2.2–3.05 ml/kg/min reported in our systematic review of publications reporting exercise capacity, including in those with and without CLD. These observations suggest that functional lung impairment is an important determinant of low exercise capacity needing differentiation from children who had a CLD diagnosis in infancy. Indeed, only 44% in the POLD group and 25% in the pPRISm group had CLD. Some previous studies including those born extremely preterm have not reported impaired exercise capacity,^26^ and our systematic review only showed differences of ~10% for peak VO_2_ when the BPD and term‐born groups were compared.^5^ This is likely to be due to the extremely preterm‐born groups including those with compromised lung function as well as a significant proportion with lung function within normal limits. Thus, by classifying our preterm group using *current* decreased lung function into those with obstructive airway disease and pPRISm groups, we were able to demonstrate clear differences for exercise capacity between the groups suggesting that current lung function assessment is likely to be more informative than a historical diagnosis such as BPD. In contrast to previous reports,^27^, ^28^ we did not find any evidence of exercise‐induced bronchoconstriction in the preterm (in particular the POLD) groups. However, the response to postexercise bronchodilator administration was impressive in the POLD children, with greater than threefold greater increase in FEV_1_ compared to controls suggesting a potential population to target therapies. Radiographic abnormalities have been previously reported especially in those who BPD in infancy showing emphysematous changes on high‐resolution computed tomography (HRCT).^23^ Since we identified children with obstructive lung based on current lung function, it is likely that this group will have significant lung microstructural abnormalities identifiable by HRCT or hyperpolarised inert gas magnetic resonance imaging (MRI) scanning.

The children with both obstructive lung disease and pPRISm had greater, but contrasting, lung function deficits compared to controls. The POLD group had greater air trapping or hyperinflation when compared to the control groups as shown by the increased RV/TLC ratios, consistent with previous findings.^29^, ^30^ Functional residual capacity (FRC), residual volume (RV), and RV/TLC but not TLC were increased in the POLD group when compared to the preterm controls suggesting a greater degree of hyperinflation in these children. The hyperinflation, particularly in those with increased RV/TLC ratio, is likely to impact exercise capacity as shown in other disorders such as cystic fibrosis.^31^ The hyperinflation, taken together with the marked response to postexercise bronchodilator, suggests another potential, more targeted intervention by identifying preterm‐born children with obstructive airway disease using outpatient‐based spirometry. A trial of a combination of inhaled corticosteroids and long acting beta2 agonists inhaler therapy is not unreasonable as recently shown,^6^ although the optimal treatment has yet to be identified. Indeed, preterm children, particularly boys, appear to perform less moderate to vigorous activity compared to term‐born children,^32^ thus any impairments may not be noticed. Alternatively, there is the possibility that impaired exercise capacity may actually be driving a more sedentary lifestyle in preterm groups.^33^ It is likely that treatment will need to be administered in association with exercise programs to improve exercise capacity, given the possible habitual lack of exercise in this group of children.^28^

We classified the preterm‐born children using the LLN suggested by GLI^10^ and classified them into those with obstructive airway disease or with preserved ratio based on their spirometry measures. The concept of PRISm is fairly recent and has yet to be explored in children. However, in adults there have been links to cardiovascular morbidity, COPD, and all‐cause mortality,^11^, ^12^ thus making it potentially important to identify children with pPRISm who may be at increased risk of future morbidity and mortality as they already have significant lung function deficits in childhood, especially as current therapies to reverse these decrements are inadequate. Within the pPRISm group, we identified that some children had reduced total lung capacity, and a third had a bronchodilator response suggesting that there may be several different phenotypes within the pPRISm group which requires further in‐depth study including assessment of gas exchange, imaging, and so on, to further identify the nature of the underlying mechanisms.

### 4.1 Strengths and limitations

The strength of this study is formally assessing a large group of children with standardized techniques and focusing on those with lung function deficits. Additionally, we have explored the ideas of different phenotypes including both obstructive lung disease and the previously uncharacterized pPRISm. We used cycle ergometry which we previously showed is associated with lower peak oxygen uptake, but our children achieving similar peak heart rate suggest all reached a similar degree of exertion, thus we remain confident of our findings.

In summary, by identifying preterm‐born children with lung function at the lower limits of normal, we have shown that they have lower exercise capacity, especially if they had obstructive airway disease. Preterm‐born children with obstructive lung disease also had worse symptoms, increased hyperinflation, increased FE_NO_, and had particularly marked response to postexercise bronchodilator administration. The increased FE_NO_ levels suggest possible continuing inflammatory processes and the post‐exercise bronchodilator response suggests reversible airway obstruction that is likely to be responsive to treatment. pPRISm needs to be studied in greater detail with longer term follow‐up to determine if it is also associated with longer term cardiorespiratory morbidity and all‐cause mortality.

## AUTHOR CONTRIBUTIONS

Sailesh Kotecha conceived, designed, and set‐up the study; Michael Cousins and KH recruited participants and performed all testing; EMW provided technical support and expertise; Michael Cousins and Sailesh Kotecha were involved in the data analysis and interpretation; Michael Cousins and Sailesh Kotecha drafted the manuscript; all authors were involved in revising the manuscript and approved the final submitted version.

## CONFLICT OF INTEREST

S. K. reports securing a research grant from the Medical Research Council for this study. S. K. reports funding from HTA/NIHR, Moulton Foundation, GSK, Nutricia Foundation, and Aspire Pharma outside this study.

## Supporting information

Supporting information.

Supporting information.

## Data Availability

Anonymized data will be available in the future due to data sharing agreements in place. Please contact the corresponding author for how to access data beyond 2023. Anyone requesting the data for a specific question to be addressed can access the data after signed data access agreement.
