# Supplementary material for: Impaired exercise outcomes with significant bronchodilator responsiveness in children with prematurity‐associated obstructive lung disease
Source: Pediatr Pulmonol. 2022 Jun 14;57(9):2161–71. doi: 10.1002/ppul.26019 (PMC9546294; doi:10.1002/ppul.26019)
Supplement: Supplementary file 1 — Supporting information. [file PPUL-57-2161-s002.docx]

**Impaired exercise outcomes with significant bronchodilator responsiveness in children with prematurity-associated obstructive lung disease** **– Online Supplement**

^1^Michael Cousins, ^1^Kylie Hart, ^2^ E. Mark Williams, ^1^Sailesh Kotecha

^1^Department of Child Health, Cardiff University School of Medicine, Cardiff, United Kingdom.

^2^Faculty of Life Sciences and Education, University of South Wales, Pontypridd, United Kingdom.

**Corresponding Author:**

Professor Sailesh Kotecha

Department of Child Health

School of Medicine

Cardiff University

Heath Park

Cardiff CF14 4XN

United Kingdom

Email: [KotechaS@cardiff.ac.uk](mailto:KotechaS@cardiff.ac.uk)

Telephone: +44(0)29 20 74 4187

Fax: +44(0)29 20 74 4283

## **ONLINE METHODS**

## **Patient population**

Patients were recruited for in-depth lung function testing based on their %FEV_1_ at a screening visit. Children with %FEV_1_ ≤85% were invited as potential participants in the Respiratory Health Outcomes in Neonates (RHiNO) randomised control trial (RCT); however, definitive decision on recruitment to the RCT was based on %FEV_1_ during the in-depth lung function testing. The primary aim of screening was to identify children who were potentially eligible for recruitment to the RCT, with the secondary aim to recruit a number of preterm and term participants who could act as control subjects. However, due to there being a larger proportion of children with %FEV_1_ >85% compared to those with %FEV_1_ ≤85%, not all children in the latter group were invited. In order to ensure balance of recruitment of preterm controls over time including recruiting over different seasons and to avoid recruiting only older subjects (as recruitment started with the oldest available children), the first ten preterm-born children for each calendar month were *a priori* assigned as potential preterm controls, if their %FEV_1_ was >85%. Children with %FEV_1_ ≤85% at in-depth lung function screening were invited to enter the RCT. Due to inherent variability of spirometry, not all children with %FEV_1_ ≤85% at screening subsequently had %FEV_1_ ≤85% at the second visit. In these instances, if that participant had been within the first 10 screening visits of a calendar month (i.e., how the preterm controls were recruited for follow-up lung function testing), they completed their visit as preterm controls. They were excluded if they were not assigned as *a priori* preterm controls.

## **Withholding medication**

Participants were asked to withhold the following medications and foods prior to testing:

- Long acting ß_2_ agonists for 48 hours before visit
- Inhaled corticosteroids for 24 hours before visit
- Short-acting ß_2_ agonists for 8 hours before visit (unless symptomatic)
- Leukotriene receptor antagonists for 48 hours before the visit
- Caffeine for 24 hours before the visit
- Antihistamines for 48 hours before the visit
- Consumption of food or drink (except water) in the last hour
- Consumption of foods containing nitrate/nitrites on the day of testing

## **Spirometry**

Spirometry was performed using the MasterScreen Body and PFT systems with SentrySuite measurement software version 2.17 (Vyaire Medical, Germany). ERS/ATS guidelines for obtaining suitable spirometry were used for as a guide for performing the test and test acceptability ^1^. An explanation and a demonstration on how to perform the test were done before the child attempted the spirometry. Spirometry was performed with the child sat upright and wearing a nose-clip. They were instructed to take the biggest breath in possible, before blowing out as hard and as fast as they could. Children were vocally encouraged to continue breathing out until they appeared to have reached their residual volume. A minimum of 3 tests were performed, aiming for the intra-test criteria as per Miller et al ^1^. Spirometry was stopped once satisfactory testing was obtained or if the child did not wish to continue or was unable to perform adequate spirometry. QC was performed to ensure the correct results from all the measurements were used. Daily volume calibrations and weekly flow calibrations were performed using a three-litre syringe. Results were measured at BTSP and Global Lung Function Initiative predicted values were used to adjust for height, ethnicity, gender and age ^2^. Spirometry was repeated at 4 separated times following conclusion of the exercise test: at 5-10 minutes; 15-20 minutes; 25-30 minutes; 40-45 minutes. This was performed as outlined above. After the final post-exercise spirometry, 400 micrograms of salbutamol (Salamol, TEVA UK Limited) was given via MDI using a Volumatic spacer (GSK, UK). The salbutamol inhaler was shaken before each actuation. Children were instructed to take 10 breaths in and out after each actuation of salbutamol, ensuring the spacer’s valve clicked with each breath. Repeat spirometry was performed 15 minutes after administration of the salbutamol, as described as above.

## **Skin prick testing**

Skin prick testing was performed using Multi-Test PC lancets (Lincoln Diagnostics, USA). A Dipwell Tray (Lincoln Diagnostics, USA) was pre-prepared with the following allergens: cat dander; dermatophagoides pterynyssinus; grass mix; dog dander; aspergillus fumigatus; and cladosporium herbarum; as well as a positive histamine control and a negative control (Immunotek, Spain). The procedure was explained to the child and their forearm was cleaned gently with water, after ensuring the skin was free from eczema or any similar skin conditions. The Multi-Test PC lancet was inserted into the Dipwell Tray ensuring all touch-posts were coated with allergen solution. The lancet was slowly removed from the tray, and gently applied to the skin. Following one second of gentle pressure, the lancet was pressed firmly onto the skin with gentle rotation of the lancet device up and down and side to side before removal. Successful application left the imprints of the touch posts on the skin. Any excess allergen fluid on the skin was gently removed with tissue paper ensuring no cross-contamination of sites. A timer was set for 15 minutes. Children were encouraged not to scratch if the arm got itchy. After 15 minutes the arm was inspected for any wheals that developed; the raised aspects of the wheals were drawn around with pen and tape was used to lift the pen mark and stuck to a data sheet. A ruler was then used to measure the widest diameter of any of the wheals. A test was deemed positive if the wheal was greater than 3 mm, along with a positive histamine control test.

## **Fractional exhaled nitric oxide (FE_NO_)**

FeNO was performed using an exhaled nitric oxide analyser (NIOX VERO, Circassia, UK). Children were instructed in its use prior to performing test. The device required a warm-up period after being switched on, following which a warning would be issued if the sensor or breathing handle were out of date. Providing there were no issues with the above, the child would breathe in deeply through a filter applied to the breathing handle before exhaling at a steady rate and pressure until the test was complete. The child would have to breathe out for 10 seconds in total, although if the child had difficulty exhaling at the required speed for this duration, a shorter test of 6 seconds was performed. An animation was used to help the children achieve the desired flow rate, consisting of blowing a cloud from one side of the screen to the other, without letting it drop off the screen or fly too high. The child performed 2 tests. Both results were documented, and the highest FeNO level was used in analysis.

## **Body plethysmography**

Body plethysmography was performed using the MasterScreen Body system with SentrySuite measurement software version 2.17 (Vyaire Medical, Germany). Testing was performed with reference to the guidelines on static lung volume testing from ERS ^3^. In summary, children were seated in the body box and the door closed. A delay of two minutes following door closure allowed for pressure and thermal equalisation. Testing was performed with the child sat upright, wearing a nose clip and holding their cheeks. Following an initial period of tidal breathing to obtain airway resistance measurements, children then breathed against a closed shutter to obtain an intrathoracic gas volume (functional residual capacity) measurement followed by a paired expiratory vital capacity manoeuvre. A minimum of 5 repeatable resistance loops and a minimum of 3 repeatable FRC/VC measurements were obtained. Results were standardised against Global Lung Function Initiative reference values ^4^. A box calibration (in addition to the previously described volume/flow calibrations) for Tau verification (box seal) and shift volume was performed each day prior to testing, in line with the manufacturer’s instructions.

## **Cardiopulmonary exercise testing**

Cardiopulmonary exercise testing was performed on a Pediatric Cycle Ergometer (Lode, Netherlands) linked to a Masterscreen CPX system (Vyaire Medical, Germany). Children wore a fitted facemask and respiratory parameters were measured using a turbine and gas sampling tube. Data were recorded in a breath-by-breath exercise programme on JLab version 5.72 (Vyaire Medical, Germany). Heart rate was recorded using a Polar H10 heart rate sensor (Polar, UK). Oxygen saturations were monitored with a Nellcor oxygen saturation monitor (Medtronic, USA). A ramp protocol was devised to facilitate the exercise testing. This involved 1 minute of baseline measurements at rest, 3 minutes of minimally-loaded cycling (7 Watts), then at an increasing rate of 1 Watt every 6 seconds (10 Watts per minute). The child was vocally encouraged to continue exercise until they could no longer consistently maintain cadence >60 rpm, with increasing encouragement as the load got higher. Perceived exertion rating was obtained every 3 minutes and at the point the child could no longer continue. 2 minutes of minimally loaded pedalling concluded the test. A maximal test was considered if two of the following criteria were achieved: Respiratory Exchange Ratio (RER) >1.00; heart rate (HR) ≥80% predicted (220 bpm – age); ≥9/10 on OMNI scale (pictorial scale for rating of perceived exertion) ^5^; peak oxygen uptake (V̇O_2_) plateau based on visual analysis. Minute ventilation, peak O_2_ uptake and CO_2_ production results were averaged from the last 15 seconds of peak exercise. Maximum load, heart rate and respiratory rate were the highest recorded value at the peak of exercise. Ventilatory reserve was calculated by the following equation: 1-(minute ventilation/maximal voluntary ventilation)*100, where MVV = FEV_1_ x 35 ^6^. An automated volume calibration and gas analyser calibration were performed on each day of testing, in line with manufacturer’s instructions.

**REFERENCES**

1. Miller MR, Hankinson J, Brusasco V, Burgos F, Casaburi R, Coates A, Crapo R, Enright P, van der Grinten CP, Gustafsson P et al. 2005. Standardisation of spirometry. Eur Respir J. 26(2):319–338.

2. Quanjer PH, Stanojevic S, Cole TJ, Baur X, Hall GL, Culver BH, Enright PL, Hankinson JL, Ip MS, Zheng J et al. 2012. Multi-ethnic reference values for spirometry for the 3-95-yr age range: The global lung function 2012 equations. Eur Respir J. 40(6):1324–1343.

3. Wanger J, Clausen JL, Coates A, Pedersen OF, Brusasco V, Burgos F, Casaburi R, Crapo R, Enright P, van der Grinten CPM et al. 2005. Standardisation of the measurement of lung volumes. Eur Respir J. 26(3):511-522.

4. Hall GL, Filipow N, Ruppel G, Okitika T, Thompson B, Kirkby J, Steenbruggen I, Cooper BG, Stanojevic S. 2021. Official ers technical standard: Global lung function initiative reference values for static lung volumes in individuals of european ancestry. Eur Respir J. 57(3):2000289.

5. Barkley JE, Roemmich JN. 2008. Validity of the caler and omni-bike ratings of perceived exertion. Med Sci Sports Exerc. 40(4):760-766.

6. Joshi S, Powell T, Watkins WJ, Drayton M, Williams EM, Kotecha S. 2013. Exercise-induced bronchoconstriction in school-aged children who had chronic lung disease in infancy. J Pediatr. 162(4):813-818 e811.
